# Comparison of long-term functional results between standard supracricoid laryngectomy and modified technique with sternohyoid muscle^☆^^☆☆^

**DOI:** 10.1016/j.bjorl.2018.02.007

**Published:** 2018-03-24

**Authors:** Selçuk Güneş, Kadir Serkan Orhan, Bora Başaran, Mehmet Çelik, Erkan Kıyak

**Affiliations:** a Istanbul University, Faculty of Medicine, Department of Otolaryngology Head and Neck Surgery, Istanbul, Turkey; b Bakırköy Dr Sadi Konuk Research and Teaching Hospital, Department of Otolaryngology Head and Neck Surgery, Istanbul, Turkey

**Keywords:** Modified supracricoid laryngectomy, Cancer, Larynx, Voice quality, Decanulation, Laringectomia supracricoide modificada, Câncer, Laringe, Qualidade de voz, Decanulação

## Abstract

**Introduction:**

Laryngeal cancer is the most common cancer of the upper respiratory tract. The main methods of treatment included surgery (partial laryngectomy and total laryngectomy) and radiation therapy. Laryngeal dysfunction is seen after both treatment modalities.

**Objective:**

The aim of the study is to compare postoperative functional results of the standard supracricoid partial laryngectomy technique and a modified supracricoid partial laryngectomy technique using the sternohyoid muscle.

**Methods:**

In total, 29 male patients (average years 58.20 ± 9.00 years; range 41–79 years) with laryngeal squamous cell carcinoma who underwent supra cricoid partial laryngectomy were included. The patients were divided into two groups in terms of the surgical techniques. In Group A, all patients underwent standard supracricoid partial laryngectomy technique between January 2007 and November 2011. In Group B, all patients underwent modified supracricoid partial laryngectomy between August 2010 and November 2011. Fiberoptic endoscopic evaluation of swallowing test, short version of the voice handicap index scores, and the MD Anderson dysphagia inventory, the time of oral feeding and the decanulation of the patients after surgery of each groups were compared.

**Results:**

The mean maximum phonation time was 8.68 ± 4.21 s in Group A and 15.24 ± 6.16 s in Group B (*p* > 0.05). The S/Z (s/s) ratio was 1.23 ± 0.35 in Group A and 1.08 ± 0.26 in Group B (*p* > 0.05); the voice handicap index averages were 9.86 ± 4.77 in Group A and 12.42 ± 12.54 in Group B (*p* > 0.05); the fiberoptic endoscopic evaluation of swallowing test averages were calculated as 12.73 ± 3.08 in Group A and 13.64 ± 1.49 in Group B (*p* > 0.05). In the MD Anderson dysphagia inventory, evaluation of swallowing, the emotional, physical, and functional scores were 29.21 ± 4.11, 32.21 ± 6.85, and 20.14 ± 2.17 in the Group B, and 29.20 ± 2.54, 32.4 ± 4.79, and 19 ± 1.92 in Group A, respectively.

**Conclusion:**

Although there is no statistical difference in functional outcome comparisons, if rules are adhered to in preoperative patient selection, modified supracricoid partial laryngectomy can be applied safely and meaningful gains can be achieved in functional outcomes.

## Resumo

**Introdução:**

O câncer laríngeo é o câncer mais comum do trato respiratório superior. Os principais métodos de tratamento incluem cirurgia (laringectomia parcial e laringectomia total) e radioterapia. A disfunção laríngea é observada em ambas as modalidades de tratamento.

**Objetivos:**

Comparar os resultados funcionais pós-operatórios da técnica de laringectomia padrão supracricoide e a técnica de laringectomia supracricoide modificada com o uso do músculo esterno-hióideo.

**Método:**

Foram incluídos 29 pacientes do sexo masculino (média de 58,20 ± 9,00 anos, intervalo de 41 a 79) com carcinoma espinocelular de laringe submetidos à laringectomia supracricoide parcial. Os pacientes foram divididos em dois grupos em termos de técnicas cirúrgicas. Todos os pacientes do Grupo A foram submetidos à laringectomia padrão supracricoide entre janeiro de 2007 e novembro de 2011. No Grupo B, todos os pacientes foram submetidos à laringectomia supracricoide modificada entre agosto de 2010 e novembro de 2011. A avaliação endoscópica da deglutição por fibra ótica, os escores da versão curta do *Voice Handicap Index* e do *MD Anderson Dysphagia Inventory*, o tempo de alimentação oral e a decanulação dos pacientes foram comparados após a cirurgia em cada grupo.

**Resultados:**

A média do tempo máximo de fonação foi de 8,68 ± 4,21 segundos no Grupo A e 15,24 ± 6,16 segundos no Grupo B (*p* > 0,05). A razão S /Z (seg/seg) foi de 1,23 ± 0,35 no Grupo A e 1,08 ± 0,26 no Grupo B (*p* > 0,05); as médias do *Voice Handicap Index* foram 9,86 ± 4,77 no Grupo A e 12,42 ± 12,54 no Grupo B (*p* > 0,05); as médias da avaliação endoscópica da deglutição por fibra ótica foram calculadas como 12,73 ± 3,08 no Grupo A e 13,64 ± 1,49 no Grupo B (*p* > 0,05). Na avaliação da deglutição pelo *MD Anderson Dysphagia Inventory*, os escores emocional, físico e funcional foram 29,21 ± 4,11, 32,21 ± 6,85 e 20,14 ± 2,17 no Grupo B e 29,20 ± 2,54, 32,4 ± 4,79 e 19 ± 1,92 no Grupo A, respectivamente.

**Conclusão:**

Embora não haja diferença estatística nas comparações de resultados funcionais, se as regras forem respeitadas na seleção pré-operatória do paciente, a laringectomia supracricoide parcial modificada pode ser aplicada com segurança e ganhos significativos podem ser alcançados em termos de resultados funcionais.

## Introduction

Laryngeal cancer is the most common cancer of the upper respiratory tract. The main methods of treatment include surgery (partial laryngectomy and total laryngectomy) and radiation therapy. Laryngeal dysfunction is a consequence of both treatment modalities. Partial laryngectomies afford preservation of three main functions of the larynx; swallowing, respiration and phonation. Different horizontal partial laryngectomy modalities (supracricoid laryngectomy or supraglottic laryngectomy) and reconstruction techniques are used depending on tumor extension.^1^, ^2^, ^3^, ^4^, ^5^

Supracricoid partial laryngectomy (SCPL), which is an alternative technique to total laryngectomy, was first described by Meyer and Rieder in 1959.^1^ The aim of this technique is to remove the tumor without using permanent tracheostomy and to preserve swallowing and speech functions.^2^, ^3^

SCPL could be performed both in supraglottic tumors with limited thyroid cartilage invasion, lack of vocal cord mobility and vocal cord involvement, and glottic tumors that have involvement of the anterior commissure, and lack of vocal cord mobility.^4^ Contraindications to this technique include subglottic invasion greater than 10 mm in the anterior aspect, cricoarytenoid joint involvement, interarytenoid space involvement, extralaryngeal spread, hyoid bone invasion, and low pulmonary capacity.^5^ The most important handicap of this technique is deterioration of laryngeal function.^6^, ^7^ Although SCPL is an organ-preserving procedure, the poor voice quality and inability to decanulate are the other common complications.^8^, ^9^, ^10^

**Figure 1:**
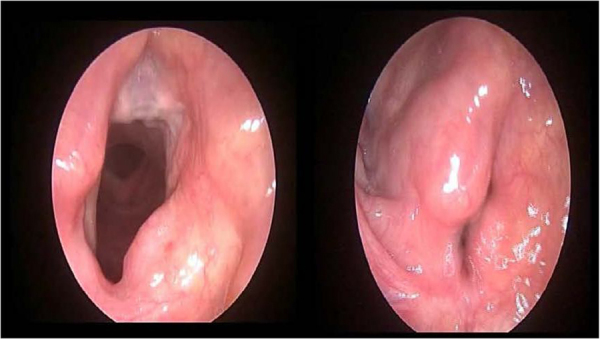
MSCPL postoperative second year.

After SCPL became popular, the functional results were published and surgeons tried to reduce the problems mentioned above. The three main topics of these quests include improving voice quality, reducing aspiration, and reducing decanulation problems. Garozzo et al. described the modified technique using the sternohyoid muscle in 2010. In this technique, after performing the SCPL, the sternohyoid muscle is transformed into a muscle tube formation and is sutured to the vocal process of the arytenoid cartilage. In this way, the aim is to reconstruct the sphincter function of the larynx, which improves voice and swallowing functions. The fascia of the muscle should be preserved in order to prevent necrosis of the muscle in the long-term period^11^ (Fig. 1). Therefore, in the literature, a few studies explored the comparison of functional results of standard SCPL (SSCPL) and modified SCPL (MSCPL) using the sternohyoid muscle. Lack of adequate knowledge about this topic legitimized the present study. We address this topic in the present study. We explored the comparison of the functional results, feeding time, decanulation time, and complications of the SSCPL and the MSCPL using the sternohyoid muscle.

The aim of the present study is to compare MSCPL with standard SCPL technique with regard to functional results.

## Methods

We retrospectively reviewed data collected from January 2007 to November 2011 on patients treated in the Department of Otolaryngology, Head and Neck Surgery, in our hospital. The study was conducted in accordance with the principles of the Declaration of Helsinki, applicable regulatory requirements and Good Clinical Practices. The protocol for this study was approved by the hospital's local ethics committee (ethical committee n° 2012/448–1003).

All patients underwent microlaryngoscopy for laryngeal biopsy and direct larynx evaluation under general anesthesia. Patients were excluded if they had any of the following characteristics: second primary cancer, distant metastases, non-squamous cell carcinoma, salvage surgeries, history of previous cancer surgeries, and history of previous radiotherapy due to laryngeal carcinoma. All of these patients were men aged between 41 and 79 years; the mean age ± SD was 58.20 ± 9.00 years. All patients underwent surgery under general anesthesia by the same surgeon. The American Joint Committee on Cancer staging system^12^ was used for reporting all patients’ T stage, nodal status and distant metastasis.

### Surgical technique

In Modified Surgical Technique partial laryngectomy (MSCPL), the skin flap is elevated and sternohyoid muscle is identified on the anterior compartment of neck. The muscle is separated from the hyoid bone with electrocautery and dissected through the thyroid cartilage. The muscle fascia is preserved in order to avoid muscle necrosis. After performing the standard SCPL, the sternohyoid muscle is reshaped as a tube-shaped structure and sutured to the vocal process of the arytenoid cartilage over the cricoid cartilage. The anterior commissure and neocord were shaped and cricohyoidopexy was completed.

Neck dissection was performed on all of the patients at the time of partial laryngectomy. The patients were divided into two groups in terms of the surgical techniques selected. In Group A, all patients underwent SSCPL between January 2007 and November 2011. In Group B, all patients underwent MSCPL between August 2010 and November 2011.

All patients of both groups were evaluated using Fiberoptic Endoscopic Evaluation of Swallowing (FEES), short version of the Voice Handicap İndex (VHI),^13^ and the MD Anderson Dysphagia Inventory (MDAID)^14^ at least 6 months postoperatively.

We did not apply any decongestion or topical anesthetic agent before FEES. Two researchers who were blinded to the patients’ data made an endoscopic examination through the nasal cavity. The larynx, hypopharynx, proximal trachea, and esophagus were visualized using a fiberoptic endoscope attached to a video recording system (KARL STORZ GmbH & Co. KG, Germany). Patients were fed 5 mL of yoghurt to evaluate swallowing. The tip of the endoscope was kept at the end of the soft palate during swallowing, and then inserted to the vocal fold level to evaluate tracheal aspiration after swallowing. Each researcher scored the aspiration independently in accordance with the scoring system of Schindler et al.^15^ The mean value of both measurements made by each researcher was used in order to avoid bias among the researchers.

The Voice Handicap Index (VHI), the Turkish version of which was validated by Kılıç MA et al.,^13^ was used to assess the patients’ voice quality, speech, and voice intelligibility, and the effects of voice impairment on the patient. The questionnaire form was completed by the patients.

The MDAID,^14^ which has been translated into Turkish but not yet validated, was used to assess variables such as the effect of the operation on the swallowing function of the patient, the effect of changed eating skills on social life, and the patient's self-esteem. The questionnaire form was completed by the patients.

FEES, short version of the VHI, and the MD Anderson Dysphagia Inventory (MDAID), postoperative complications rate and the time of oral feeding and the decanulation of the patients after surgery of each group were compared.

### Statistical analysis

IBM SPSS statistics for Windows, Version 21.0 (Armonk, NY: IBM Corp. Released 2013.) was used for all statistical analyses. Descriptive statistics (means and standard deviation, medians with inter quartile range) were derived. The significance of each intergroup difference was analyzed using Student's *t*-test, Mann–Whitney *U* test and the significance of any difference in median values was explored with the aid of the Chi-square test. A *p*-value < 0.05 was considered to reflect statistical significance.

## Results

In total, 71 patients with laryngeal squamous cell carcinoma who underwent SCPL were operated with either SSCPL or MSCPL techniques. All 71 subjects were included in the complication analysis. Forty-two patients were excluded for functional analysis (21 could not be contacted, 9 received adjuvant radiotherapy for advanced-stage disease, 6 underwent total laryngectomy due to disease recurrence, 4 could not be decanulated, and 2 underwent gastrostomy). Finally, a total of 29 patients were evaluated for functional analysis. Group characteristics are summarized in Table 1. Functional tests were performed on 15 patients in Group A and 14 patients in Group B. The follow-up time was 38.2 ± 16.55 months (range 21–71 months) in Group A and 20.78 ± 5.84 months (range 14–31 months) in Group B.

**Table 1:** Demographic data of groups.

	MSCPL	SSCPL	*p*-value^a^
Operation age	58.50 ± 8.85	57.93 ± 9.45	0.997
Clinical T stage^a^	13/1	13/2	0.853
Clinical *N* stage	0.7 ± 0.26	0 ± 0	0.000
Neck dissection	14	15	0.211
Gender	14 male	15 male	

Clinical description of the patients, *p* > 0.05 (Chi-Square Test).

a Initial tumors (T_1_ + T_2_)/advanced tumors (T_3_ + T_4_).

The mean oral intake time was 19.8 ± 12.8 days in group A and 26.14 ± 24.09 days in Group B. No statistically significant difference was found between the two groups (*p* > 0.05). The mean decanulation time was 23.13 ± 22.6 days in Group A and 40.14 ± 23.77 days in Group B (*p* > 0.05). The mean maximum phonation time was 8.68 ± 4.21 s in Group A and 15.24 ± 6.16 s in Group B (*p* > 0.05).

The S/Z (s/s) ratio was 1.23 ± 0.35 in the Group A and 1.08 ± 0.26 in Group B (*p* > 0.05); the VHI averages were 9.86 ± 4.77 in Group A and 12.42 ± 12.54 in Group B (*p* > 0.05); the FEES averages were calculated as 12.73 ± 3.08 in Group A and 13.64 ± 1.49 in Group B (*p* > 0.05). None of the results was statistically significant (Table 2).

**Table 2:** Postoperative functional parameters of groups.

	Group A (*n* = 15)	Group B (*n* = 14)	*p*-value^a^ (>0.05)
OIT (day)	19.8 ± 12.8	26.14 ± 24.09	0.680
DT (day)	23.13 ± 22.6	40.14 ± 23.77	0.381
FEES	12.73 ± 3.08	13.64 ± 1.49	0.160
VHI	9.86 ± 4.77	12.42 ± 12.54	0.232
MFS (sn)	8.68 ± 4.21	15.24 ± 6.16	0.413
S/Z ratio	1.23 ± 0.35	1.08 ± 0.26	0.290

OIT, oral intake time; DT, decanulation time; MSCPL, modified supracricoid partial laryngectomy; SSCPL, standard supracricoid partial laryngectomy; FEES, fiberoptic endoscopic evaluation of swallowing; VHI, voice handicap index; MFT, maximum phonation time.

a *χ*^2^ test.

In the MDAID evaluation of swallowing, the emotional, physical, and functional scores were 29.21 ± 4.11, 32.21 ± 6.85, and 20.14 ± 2.17 in the Group B, and 29.20 ± 2.54, 32.4 ± 4.79, and 19 ± 1.92 in Group A, respectively. There was no statistical difference between the two groups in the statistical evaluation (*p* > 0.05) (Table 3).

**Table 3:** MD Anderson dysphagia inventory of groups.

	Group B (*n* = 14)	Group A (*n* = 15)	*p*-value^a^
Emotional	29.21 ± 4.11	29.21 ± 2.63	0.93
Physical	32.21 ± 6.85	32.28 ± 4.95	0.93
Functional	20.14 ± 2.17	19.07 ± 1.97	0.104

a Mann–Whitney *U* test (*p* > 0.05).

The great difference in the decanulation time was mainly due to the long follow-up period with canulae in two patients with neck emphysema, and one patient with neck infection in Group B (Table 4).

**Table 4:** Comparison of postoperative complications in two groups.

	Group A (*n* = 48)	Group B (*n* = 23)
Decanulation problem	4/48 (8.33%)	3/23 (13.04%)
Gastrostomy	2/48 (4.16%)	0
Neck emphysema	0	2/23 (98.6%)
Neck infection	0	1/23 (4.34%)

## Discussion

SCPL is an alternative technique to total laryngectomy that aims to preserve the main laryngeal functions. The basic philosophy of the technique is that at least one functional unit is protected and swallowing and speech functions can be achieved without permanent tracheotomy. Following the acceptance of the technique by surgeons, research has focused on the problems of aspiration, voice quality, and decanulation, which are related to the failure to achieve satisfactory sphincter function. In literature, various SCPL techniques were described. However, data on functional results of these various SCPL techniques remains sparse. Lack of data on factors, the success of the various SCPL techniques hinders the resolution of several controversial issues. Identification of such factors remains a current issue in otorhinolaryngology.

**Figure 2:**
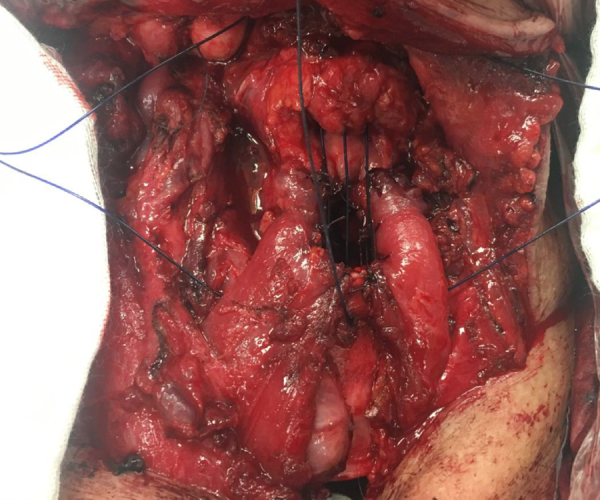
Surgical view of the MSCPL.

As a result of these investigations, the technique of hanging sinus pyriformis described by Benito et al.^16^; the technique of homologous cartilage placement instead of the arytenoid, which has to be resected, described by Garozzo et al.^17^; the corniculate reconstruction technique described by Loyo et al.^18^; and protecting the posterior 1/3 part of the vocal cord on the uninvolved side technique described by Adamopoulos et al.^19^; and finally, the technique of sternohyoid muscle reconstruction as described by Garozzo et al.^11^ were developed (Fig. 2).

The oral intake time is an important parameter that gives an idea of the sphincter function of the larynx. In literature, the time to begin oral feeding differs, the oral intake time was ranging from 3 to 45 days after surgery.^16^, ^20^, ^21^, ^22^ Allegra et al.^3^ and Yu et al.^23^ showed that oral intake time was significantly shorter in the MSCPL group than in the SSCPL group. In contrast to these results, we found no significant difference between the two groups in our study.

Decanulation time is another important parameter in the evaluation of sphincter function, allowing evaluation of the respiratory function of the larynx. In the study of Allegra et al.^3^ and Yu et al.,^23^ the decanulation time was significantly shorter in the MSCPL group than in the SSCPL group. However, no significant difference was found between the two groups in the present study.

Phonation is another important function of the larynx. Many methods can be used to evaluate phonation. These may be questionnaires that evaluate patients’ quality of life, such as the voice handicap index, or objective parameters such as the maximum phonation time, S/Z ratio and INFVo (intelligibility, noise, fluency, and voicing) scale. Allegra et al.^3^ evaluated phonation function using VHI and INFVo in their study and concluded that voice quality was better with MSCPL than SSCPL using both methods. However, they cannot achieve significant results in VHI functional, emotional, and total scores. In the present study, the phonation function was assessed by VHI short version and maximum phonation time S/Z ratio. Although the mean maximum phonation time and the mean S/Z ratio were better in the MSCPL group, there was no significant difference between the two groups. In addition, no significant results were obtained in VHI scores between the two groups.

A variety of factors affecting dysphagia after SCL are discussed in the literature; type of reconstruction, swallow training by swallow therapist, radiation, arytenoid resection, extended procedures and age.^24^ Also, in the literature, there have been limited studies investigating the dysphagia status in patients with supracricoid laryngectomy. Swallow dysfunction in patients with laryngeal cancer can be seen in preoperative and postoperative time. Therefore, it is difficult to compare results. One can conclude that to date there is no standard protocol to describe and measure the swallow function. The study of Yücetürk et al.^20^ has reported that this could indicate that movements of the neoglottis are close to normal and patients with SCPL have effective and near normal swallowing, tolerating a near normal diet. In evaluation of swallowing function in patients undergoing SCPL, MD Anderson Dysphagia Inventory for self-assessment, FEES scoring, and the Dysphagia Outcome and Severity Scale (DOSS)^25^ for objective evaluation can be used. Yu et al.^23^ noted that the swallowing function in the MSCPL group was better than the SSCPL group as a result of their evaluation with DOSS. We used both FEES and MDAID methods to evaluate swallowing function. In swallowing evaluation, although the FEES scores were better in the MSCPL group, no statistically significant difference was found because of the high standard deviation. However, in the MDADI evaluation, there was no difference between the two groups in all three sections (emotional, functional, and physical). On the other hand, in the study of Yu et al.,^23^ recurrent aspiration pneumonia in one patient and NGT dependency in another patient from the SSCPL group were reported. In both studies, the absence of patients who were dependent on NGT or who had to undergo open gastrostomy in the MSCPL group could be interpreted as achieving better swallowing function with MSCPL. In the present study, one patient from the SSCPL group had to undergo open gastrostomy because of NGT dependency.

In the present study, six patients (two from the MSCPL group and 4 from the SSCPL group) could not be decanulated. Allegra et al.^3^ did not discuss the decanulation rate, but Yu et al.^23^ showed that 6 patients (33.3%) in the SSCPL group and 1 patient (4.3%) in the MSCPL group could not be decanulated, which is similar to our results.

In addition, although Allegra et al.^3^ stated that they had no postoperative complications in either the SSCPL or MSCPL groups, except for the detachment of one of the two sternohyoid muscle in two cases, in contrast, Yu et al.^23^ reported that three patients developed postoperative wound infections and delayed wound healing in the MSCPL group and one patient had the same complications in the SSCPL group. In the present study, internal jugular venous rupture and subcutaneous emphysema were observed in two patients in the MSCPL group, which were not seen previously in the SSCPL group. Both complications in the present study were due to sternohyoid muscle necrosis. These results may be due to the fact that neck dissection was applied to all patients in our study, different from the study of Allegra et al.^3^ Similarly, Yu et al.^23^ reported three wound infections and delayed healing in 23 patients who underwent MSCPL, and 11 of 23 patients who underwent neck dissection.

In the literature, there are few studies comparing the functional results of various SCPL techniques; the reported studies were mostly compared the oncologic results. The study of Leszczyńska et al.^26^ have reported that modified SCL was superior in earlier extubation, with significantly lower need for temporary and permanent tracheotomy and better swallowing. The cited authors concluded that the voice was altered in all analyzed SCL patients, the modified SCL operation seem to be superior to standard SCL in terms of voice quality.

Although we achieved interesting results, there are limitations to the present study. The size of the study population may also be a limitation. Other limitations of the present study include the lack of a validated MDAID questionnaire and the lack of randomization. In addition, cultural conflicts could have influenced the results. Likewise, the oncologic outcomes were not been investigated. If the study was a prospective randomized study with larger numbers of patients, the study would be more valuable.

In the present study, we compared the functional results of two techniques. We did not observe the differences in the functional results of each group. Even though many different surgical approaches were described in literature, the gold standard technique of SCPL has not yet been determined.

## Conclusion

In conclusion, our data suggested that the functional results of two SCPL techniques were similar. However both techniques (SSCPL and MSCPL) can be applied safely and meaningful gains can be achieved in functional outcomes. Further studies with larger numbers of patients are needed to compare the functional and oncological results of various SCPL techniques.

## Conflicts of interest

The authors declare no conflicts of interest.
